# The Endoplasmic Reticulum Role in the Plant Response to Abiotic Stress

**DOI:** 10.3389/fpls.2021.755447

**Published:** 2021-11-18

**Authors:** Sofía Reyes-Impellizzeri, Adrian A. Moreno

**Affiliations:** Centro de Biotecnología Vegetal, Universidad Andrés Bello, Santiago, Chile

**Keywords:** endoplasmic reticulum quality control (ERQC), endoplasmic reticulum associated degradation (ERAD), unfolded protein response (UPR), chaperone, abiotic stress

## Abstract

The endoplasmic reticulum (ER) is the organelle where one third of the proteins of a cell are synthetized. Several of these proteins participate in the signaling and response of cells, tissues, or from the organism to the environment. To secure the proper synthesis and folding of these proteins, or the disposal of unfolded or misfolded proteins, the ER has different mechanisms that interact and regulate each other. These mechanisms are known as the ER quality control (ERQC), ER-associated degradation (ERAD) and the unfolded protein response (UPR), all three participants of the maintenance of ER protein homeostasis or proteostasis. Given the importance of the client proteins of these ER mechanisms in the plant response to the environment, it is expected that changes or alterations on their components have an impact on the plant response to environmental cues or stresses. In this mini review, we focus on the impact of the alteration of components of ERQC, ERAD and UPR in the plant response to abiotic stresses such as drought, heat, osmotic, salt and irradiation. Also, we summarize findings from recent publications looking for a connection between these processes and their possible client(s) proteins. From this, we observed that a clear connection has been established between the ERAD and UPR mechanisms, but evidence that connects ERQC components to these both processes or their possible client(s) proteins is still lacking. As a proposal, we suggest the use of proteomics approaches to uncover the identity of these proteins and their connection with ER proteostasis.

## Introduction

Plants are exposed to continuous environmental changes such as water bioavailability, salinity, temperature, and irradiation that require a fast and dynamic response from the plant to survive under these adverse conditions. Usually, this response involves changes of gene expression, and concomitantly, changes on the plant cell proteome (Zhu, 2016). It is estimated that more than one third of plant cell proteins are synthesized and folded at the endoplasmic reticulum (ER) (Chen et al., 2005). To keep this process as efficient as possible, at the lumen of the ER, several chaperones and co-chaperones assist the folding of unfolded or misfolded proteins (Strasser, 2018). Moreover, a specific subset of chaperones and co-chaperones participate in the folding of glycoproteins in a process known as ER quality control (ERQC); a mechanism that relies on the modification of an N-glycan structure present on N-glycosylated proteins. As any process, the synthesis and folding of proteins at ER considers the production and persistence of misfolded proteins, which are degraded by a specific group of proteins in a mechanism termed ER associated degradation (ERAD) (Strasser, 2018). Under certain conditions, these two mechanisms —ERQC and ERAD— are surpassed by the accumulation and persistence of unfolded and/or misfolded proteins, leading to an organellar state that triggers signaling pathways that communicate the ER with the nucleus, in order to restore the ER synthesis, folding and degradation capabilities or proteostasis; this process is known as the unfolded protein response (UPR) (Howell, 2013). The UPR consist of two main signaling branches in plants, the IRE1/bZIP60 and bZIP17/bZIP28, where the first involves the processing of the transcription factor bZIP60 messenger RNA by a membrane-bound kinase-RNAse termed IRE1 (Deng et al., 2011; Nagashima et al., 2011; Moreno et al., 2012). The second branch comprises two transcription factors, bZIP28 and bZIP17, that are cleaved in the Golgi apparatus by two serine proteases known as S1P and S2P (Liu et al., 2007a, b). All these components of the ER protein processing and signaling pathways have been associated with the plant response to abiotic stress. Nevertheless, no client(s) protein(s) have been described as responsible for the effects observed on ER chaperone, co-chaperone or signaling components mutants that exhibit sensitivity to these abiotic stress conditions. In the present review, we will focus on the chaperones, co-chaperones and signaling components that exhibit phenotypes of sensitivity or tolerance to abiotic stress conditions such as cold, heat, osmotic and salt stresses, proposing experimental approaches to understand their role and their possible client(s) protein(s).

### Endoplasmic Reticulum Quality Control Components and Abiotic Stress

BiP is a HSP70-like chaperone protein and one of the most abundant chaperones within the ER lumen. Under proteostasis it binds to the hydrophobic surface of nascent polypeptide chains incorporated into the lumen, preventing aggregation. When UPR occurs, BiP disassociates bZIP28 to transiently bind to misfolded or unfolded proteins, leading to the downstream signaling of UPR (Srivastava et al., 2013). This is possible because the luminal domain of bZIP28 contains regions of disordered conformation that recruit BiP, controlling the activation of this transcription factor but under UPR conditions, the affinity of this chaperone for these regions is lower compared to unfolded or misfolded proteins, releasing bZIP28 and promoting its activation (Srivastava et al., 2013). Although, it has been reported that a similar process occurs between BiP and IRE1 in yeasts (Pincus et al., 2010), the role of BiP on the activation of IRE1 signaling in plants as the possibility that IRE1 can bind misfolded proteins, remains to be demonstrated. BiP has been found to contribute to plant resistance in many kinds of abiotic stress, such as osmotic, heat, heavy metal, cold and salt stress. In soybean, four genes encoding BiP proteins have been described, and soyBiPD has been linked to higher drought tolerance in soybean (Valente et al., 2009) and in *Nicotiana tabacum* (Alvim et al., 2001). In maize 22 BiP-like genes have been identified, but only one of them, ZmBIPb, was found to be upregulated by ER stress induced with DTT and heat (Li et al., 2012). The possible involvement of other ZmBIPs into these and other stresses, it remains to be determined. In wheat three BiP genes have been described; TaBiP1, TaBiP2 and TaBiP3, all of them are up-regulated during drought stress (Zhu et al., 2014), nonetheless their specific involvement in different UPR-inducing conditions is yet to be determined. A study of *Lycium chinense* identifies LcBiP and reports that the overexpression of this gene on tobacco confers resistance to cadmium-induced stress (Guan et al., 2015). Another report in *Capsicum annuum* L. identified three genes encoding BiP proteins; CaBiP1, CaBiP2 and CaBiP3, where CaBiP1 overexpression in *Arabidopsis thaliana* was found to aid plant tolerance to salt, osmotic and drought stress (Wang H. et al., 2017). Recently, research in citrus species reported the presence of CsBiP1 and CsBiP2 genes and showed that under drought stress the expression of CsBiP1 is upregulated in *C. limonia*, and CsBiP2 was observed to be upregulated in *C. sinensis* seedlings under cold and osmotic stress (Guimarães et al., 2018), an interesting observation that offers a unique opportunity to study the regulation and role of BiP on different stresses. A similar observation has been made on *Solanum tuberosum*, where three candidates BiPs; StBiP1, StBiP2, and StBiP3 have been identified and only StBiP3 is upregulated under salt stress (Herath et al., 2020). Overall, these reports provide strong evidence that BiP proteins are linked with different types of abiotic stresses, nevertheless none of them identified or associated any client(s) protein(s) to the observed phenotype, so further research into client(s) of these chaperones, and the search for new BiPs in other plants, would contribute to a better understanding of the role of BiP under stress.

Calreticulin (CRT) and calnexin (CNX) are molecular chaperones that aid the proper folding of proteins in the ER. CRT is the principal Ca2 + storage/buffering protein in the ER, and it is associated with the protein folding process and Ca2 + homeostasis. On the other hand, CNX directly aids protein folding by interacting with nascent proteins and assisting the folding process. Both CRT and CNX are similar in sequence and contribute to the restoration of the proteostasis during the UPR (Garg et al., 2015). Regarding CRT and abiotic stress, it has been reported in common wheat (*Triticum aestivum L*.), that different isoforms of calreticulin have been identified and overexpressed in tobacco plants, leading plants with enhanced tolerance to abiotic stress. TaCRT1, was found to contribute to salt tolerance when overexpressed in tobacco, whereas TaCRT3 was found to enhance tobacco plants tolerance to drought stress (Jia et al., 2008; Xiang et al., 2015). Later, the study of a CRT isoform present on the D genome of wheat, named TaCRT-D, showed that overexpression of this isoform on *A. thaliana* enhances plants tolerance to salt, drought, and osmotic stress (Wang J. et al., 2017). Rice CNX (OsCNX) has been linked to higher drought and cold tolerance when this gene was overexpressed in tobacco plants (Sarwat and Naqvi, 2013). Recently, a study of CNX1 gene in *Tradescantia* clone BNL 4430 referred to as TrCNX1, showed that the expression of this gene is induced in the plant upon gamma irradiation. Moreover, authors demonstrated a protective role of this chaperone against irradiation stress (gamma and UV-B) when overexpressed in *E. coli*, but studies *in planta* are still required to understand the participation of this molecular chaperone during irradiation stress (Ha et al., 2020).

Protein disulfide isomerases (PDIs) are molecular chaperones that participate in the protein folding process by catalyzing disulfide bonds. In *A. thaliana*, 12 PDI-like proteins have been described, from these AtPDI1 to AtPDI6 plus AtPDI9, AtPDI10 and AtPDI11 are reported to be located in the ER (Yuen et al., 2013). Recently, the expression of AtPDI9 was found to increase during heat stress, and mutant plants on this gene exhibit low pollen viability and altered morphology under heat stress (Feldeverd et al., 2020). A recent study in *Medicago truncatula* identified 17 PDI coding genes, from which MtPDI1-1, MtPDI2-1, MtPDI2-2, and MtPDI4-1 were found to increase expression levels under ER stress (Meng et al., 2021). Other report on *Solanum lycopersicum* L. identified 19 PDI coding genes, where the expression of SlPDI1-1, SlPDI1-3, SlPDI1-4, SlPDI2-1, SlPDI4-1, and SlPDI5-1 was regulated by several abiotic stress and ABA treatment (Wai et al., 2020). Whether or not MtPDIs or SlPDIs participate in the plant response to abiotic stresses should be determined.

UDP-Glucose:Glycoprotein Glucosyltransferase (UGGT) is an enzyme that plays a central role on the ERQC system, since it controls the re-entry of unfolded or misfolded glycoproteins to the system through the modification of the N-glycan, present on N-glycosylated glycoproteins. It has been reported that mutants on UGGT in *A. thaliana* are sensitive to salt and heat stress (Blanco-Herrera et al., 2015). In the case of UGGT, client’s proteins are known, for example brassinosteroid insensitive 1 receptor (BRI1) and EF-Tu receptor (EFR) (Jin et al., 2007; Li et al., 2009). Interestingly, the mutant form of the BRI1 receptor, *bri1-9*, has been directly associated with sensitivity to salt stress, since plants expressing *bri1-9* showed a lower germination and grown than wild type plants exposed to salt stress (Cui et al., 2012).

### Other Endoplasmic Reticulum Chaperones and Co-chaperones Involved in Abiotic Stress

An ER-located small heat-shock protein (ER-sHSP) has been shown to act as a molecular chaperone (Mamedov and Shono, 2008), and its overexpression relieve ER stress induced by tunicamycin or DTT although its transcript is not being induced by these chemical agents (Zhao et al., 2007). Moreover, the transgenic tomato plants expressing constitutively this ER-sHSP exhibit a high salt tolerance, accumulated more osmolytes and soluble sugars, and absorbed less Na + maintaining higher levels of K + and Ca2 + compared to control plants (Fu et al., 2016). Interestingly, these plants show lower expression levels of ER molecular chaperones, even under ER stress inducing conditions and while still having tolerance against NaCl induced stress.

Sensitivity to Salt 1 (SES1) was identified in a screening looking for mutants sensitive to salt and has been found to alleviate heat stress and salt stress in plants at the same time it acts as an ER molecular chaperone (Guan et al., 2018, 2019). Mutant plants on *ses1* exhibit a higher expression of ER chaperones and other proteostasis related components under salt and heat stress conditions compared to wild type plants, but in neither case, the overexpression of this gene confers a higher tolerance to these stresses to plants (Guan et al., 2018, 2019). It also contains an ERSE-L-like element on its promoter region, which was proven to be able to bind to bZIP17 (Guan et al., 2018) and bZIP28 (Guan et al., 2019), indicating that bZIP17 and bZIP28 could be responsible for SES1 expression during salt and heat stress, respectively. An attractive proposal is that a heterodimer formed by bZIP17 and bZIP28 could potentially bind to SES1 and activate its expression under abiotic stress conditions (Guan et al., 2019).

A list of ER chaperones and co-chaperones associated to phenotypes of tolerance to abiotic stresses is depicted on Table 1.

**TABLE 1:** List of ER chaperones associated with a tolerance phenotype to abiotic stress.

Chaperone name	Isoforms related to ER stress	Origin	Subcellular localization	Related stress	Known targets	References
TaBiP	TaBiP1, TaBiP2 and TaBiP3	*Triticum aestivum*	ER*	Drought	Unknown	Zhu et al., 2014
soyBiP	soyBiPD	*Glycine max*	ER*	Drought	Unknown	Valente et al., 2009
ZmBiP	ZmBiPb	*Zea mays*	ER*	Heat	Unknown	Li et al., 2012
LcBiP	LcBiP	*Lycium Chinese*	ER*	Cadmium	Unknown	Guan et al., 2015
CaBiP	CaBiP1	*Capsicum annuum L.*	ER	Heat, salt, osmotic and drought	Unknown	Wang H. et al., 2017
StBiP	StBiP3	*Solanum tuberosum*	ER	Salt	Unknown	Herath et al., 2020
CsBiP	CsBiP1	*Citrus limonia*	ER*	Drought	Unknown	Guimarães et al., 2018
CsBiP	CsBiP2	*Citrus sinensis*	ER*	Cold and osmotic	Unknown	Guimarães et al., 2018
OsCNX	OsCNX	*Oryza sativa*	ER*	Drought	Unknown	Sarwat and Naqvi, 2013
TrCNX	TrCNX1	*Tradescantia* clone BNL 4430	ER*	Irradiation	Unknown	Ha et al., 2020
TaCRT	TaCRT3	*Triticum aestivum L.*	Cytoplasmic and nuclear compartments	Drought	Unknown	Jia et al., 2008
TaCRT	TaCRT-D	*Triticum aestivum L.*	ER*	Salt, drought, mannitol	Unknown	Wang J. et al., 2017
TaCRT	TaCRT1	*Triticum aestivum L.*	ER*	Salt	Unknown	Xiang et al., 2015
AtPDI	AtPDI9	*Arabidopsis thaliana*	ER	Heat	Unknown	Yuen et al., 2013; Feldeverd et al., 2020
SES1	SES1	*Arabidopsis thaliana*	ER	Heat and salt	Unknown	Guan et al., 2018, 2019
ER-sHSP	ER-sHSP	*Lycopersicon esculentum*	ER*	Salt	Unknown	Fu et al., 2016

*ER localization is predicted but unconfirmed experimentally.

### Endoplasmic Reticulum Associated Degradation Role on Abiotic Stress

The first ERAD component to be associated with abiotic stress was SEL1L/HRD3A, part of the complex HRD1/HRD3, since T-DNA insertional mutants on this gene showed a salt stress sensitivity phenotype in *A. thaliana* (Liu et al., 2011). Moreover, Liu et al. (2011) described that *hrd3a* mutants also exhibit sensitivity to reactive oxygen species (ROS) inducing agents like paraquat, suggesting that a possible explanation to the phenotype observed upon salt stress on these plants could be related to their lower capacity to deal with ROS. Later, another component of ERAD known as OS9, was described to interact with SEL1L and a mutant on OS9 also exhibited a phenotype of sensitivity to salt stress in *A. thaliana* (Hüttner et al., 2012).

Another component of ERAD, ubiquitin-conjugating enzyme 32 (UBC32), has been described to act as a negative regulator of plant tolerance to salt stress in *A. thaliana* (Cui et al., 2012). In this work, authors observed that *ubc32* mutant plants are more tolerant to salt stress whereas overexpressor plants are more sensitive. Moreover, Cui et al. (2012) showed that an *ubc32* T-DNA insertional mutant can restore the salt sensitivity phenotype of *bri1-9* mutant, suggesting that salt stress phenotype observed in *ubc32* mutant plants is related to BR signaling. Recently, Ahn et al. (2018) reported that UBC32 and two homologs, UBC33 and UBC34, act as negative regulators of plant response to drought stress in *A. thaliana*, reinforcing the role of ERAD components in the plant response to abiotic stresses.

### Unfolded Protein Response Signaling Under Abiotic Stress

The first association of bZIP28 with abiotic stress was reported in *A. thaliana* by Gao et al. (2008). This work indicates that bZIP28 was an important component of the plant response to heat stress and it was involved in the regulation of chaperones expression. Further work confirms the role of bZIP28 in the plant response to heat stress and connect it to the maintenance of fertility under heat stress (Zhang et al., 2017).

In the case of bZIP17, it was associated with plant response to salt stress by Liu et al. (2007b). This work indicates that T-DNA insertional mutants on bZIP17 are more sensitive to salt stress. A subsequent work explores the possibility that the overexpression of a soluble form of bZIP17 could enhance the plant tolerance to salt stress with positive results when this construct was under the control of a stress inducible promoter (Liu et al., 2008). A recent work by Ramakrishna et al. (2018), showed that bZIP17 overexpression from Finger millet (*Eleusine coracana* L.) in tobacco plants enhanced tolerance to salt, osmotic, drought and heat stress.

With regards to the IRE1/bZIP60 signaling arm of the UPR, and plant response to heat stress, it has been reported a close association (Deng et al., 2016). Also, it has been reported that bZIP60 overexpression has a positive effect in plant tolerance to salt stress (Fujita et al., 2007). Moreover, it has been recently reported that IRE1 also could play a role in plant response to salt stress, specifically in the maintenance of the root meristem under salt stress (Iwata et al., 2017). Later, Babitha et al. (2015) described that overexpression of bZIP60 from Finger millet (*Eleusine coracana* L.) in tobacco plants enhanced tolerance to salt, osmotic, drought and chemical induced ER stress. Recently, the overexpression of the spliced form of an ortholog of AtbZIP60 from the resurrection plant *Boea hygrometrica* (BhbZIP60S) on *A. thaliana*, was shown to confer tolerance to drought, tunicamycin and mannitol (Wang B. et al., 2017). A similar observation was made by Geng et al. (2018), where the overexpression of the spliced form of TabZIP60 from wheat (*Triticum aestivum*) in *A. thaliana*, confers tolerance to heat stress but no other abiotic stress was analyzed.

### Integration of Endoplasmic Reticulum Quality Control, Endoplasmic Reticulum Associated Degradation, and Unfolded Protein Response During Abiotic Stress

Since the first report of bZIP17 role on salt stress in *A. thaliana* (Liu et al., 2007b), a question regarding the regulation of ERQC components under salt stress by UPR components has been a focus of attention. Liu et al. (2007b) reports that bZIP17 regulates other target genes different from ERQC components. Later, Che et al. (2010) associated the activation of bZIP17 to heat stress and described that the soluble form of this transcription factor, under its own promoter or under a constitutive one, like CaMV 35S, can regulate the expression of several ERQC components such as BiP, CRT, CNX and PDIL under no stress conditions. Another observation from Henriquez-Valencia et al. (2015), indicates that from several ERQC components, only BiP3 is upregulated under salt stress conditions and its expression is dependent on bZIP17. These differences could be associated to the transcription factors that are induced specifically under certain abiotic stresses that can bind the promoter of ERQC components as it has been recently proposed (Ko and Brandizzi, 2021). A similar situation occurs with bZIP60, since the first report that indicate its involvement on salt stress, showed that no ERQC gene expression was altered on plants that overexpress bZIP60 under unstressed conditions (Fujita et al., 2007). A possibility is that under no stress conditions, no processing of bZIP60 by IRE1 occur, so no changes in the expression of ERQC components take place. Later, another report indicates that the overexpression of a truncated version of the unspliced form bZIP60 lacking the C-terminal portion contain a transmembrane domain conferred tolerance to salt stress but no connection between ERQC components and bZIP60 was established (Tang et al., 2012). Recently two reports indicate the up regulation of ERQC components on plants that overexpressed bZIP60 from *Eleusine coracana* L. and *Boea hygrometrica* (Babitha et al., 2015; Wang B. et al., 2017). Different scenario is associated with bZIP28 where its tolerance to heat stress has been associated with changes in expression on ERQC components (Deng et al., 2016).

With regards to the association between ERAD and UPR, the work by Li et al. (2017) established a connection between a component of ERAD, HRD3A, and each of the UPR components bZIP17, bZIP28 and bZIP60. Interestingly, the susceptible phenotype of *hrd3a* to salt stress can be compensated by the overexpression of bZIP17, bZIP28 and bZIP60. Moreover, this work shows that overexpression of these transcription factors leads to increased expression of ERQC components under salt stress conditions, suggesting a connection between the increase of ERQC components’ levels and the plant tolerance to salt stress. In agreement with these observations, a recent work by Tian et al. (2019), reports that pretreatment of plants with Tunicamycin or DTT, leads to a lower mortality rate when plants are exposed to high salt stress conditions (200 mM). Also, authors indicate an increase on the transcript level of ERQC, UPR, and ERAD components on their salt acclimation system. Furthermore, Tian et al. (2019) explore the role of bZIP17 and HRD3A on plant acclimation to salt stress, and their results showed that both genes play an important role on this process, where *bzip17* mutants exhibit an almost total compromise on plant acclimation to salt. Overall, these observations highlight the role of ERAD and UPR in the plant response to salt stress, but whether these mechanisms are related to other abiotic stresses is a question that remains.

## Future Challenges and Perspectives

One of the major questions that remains, after the significant progress that has been made on the role of the ER proteostasis in the plant response to abiotic stress, is what the clients of the ERQC mechanism are and how these are related to the plant response to this kind of stress. As far as it is known, the mutant version of the BRI1 receptor, *bri1-9*, is the only protein that has been connected to ER proteostasis and salt stress. Nevertheless, the *bri1-9* protein is not an endogenous protein, exposing the fact that no client(s) protein(s) are known to require ERQC components under abiotic stress conditions. Moreover, from all the chaperones and co-chaperones reviewed on this work (Table 1), no client(s) has been described. To identify these proteins, we propose the use of established approaches like quantitative proteomics analyses or novel ones such as proximity labeling. Indeed, on the ER proteostasis field of research, Lyu et al. (2020) has explored the differences in protein composition between *A. thaliana* ecotypes Col-0 and Ler under ER stress conditions induced by Tunicamycin, based on a previous observation that Ler ecotype plants are more susceptible to chemical induced ER stress. As a result of this approach, Lyu et al. (2020) found out that 11 proteins related to protein folding, protein degradation and vesicle trafficking were differentially accumulated on Col-0 ecotype plants suggesting that this approach could contribute to the understanding of the role of certain ER proteostasis components (ERQC, ERAD and UPR) and identify their client(s) on plant response to abiotic stresses. Another strategy would be the use of proximity labeling approach, a new technique that has been probed in plants and has demonstrated to be efficient in the identification of interacting proteins (Mair et al., 2019; Zhang et al., 2019; Cho et al., 2020). Indeed, Kim et al. (2021) reports the tracking of secretory proteins using this technique in a mouse model, an approach that consider the expression ER-localized version of a labeling protein (TurboID), suggesting that the labeling of proteins in the ER lumen is possible. We envision that the fusion of ERQC chaperones to labeling proteins will help the identification of client(s) of these ERQC chaperones under abiotic stresses such as drought, osmotic, salt and irradiation. Also, we consider that the use of multi-OMICs approaches could help to understand the role of ER proteostasis in the plant response to abiotic stress as this strategy has been demonstrated in human cells derived from patients with a particular form of amyotrophic lateral sclerosis (Straub et al., 2021). The use of these methods will help to answer this outstanding question and move forward the ER proteostasis field.

## Author Contributions

AM developed the conceptualization. SR-I and AM wrote the manuscript. Both authors contributed to the article and approved the submitted version.

## Conflict of Interest

The authors declare that the research was conducted in the absence of any commercial or financial relationships that could be construed as a potential conflict of interest.

## Publisher’s Note

All claims expressed in this article are solely those of the authors and do not necessarily represent those of their affiliated organizations, or those of the publisher, the editors and the reviewers. Any product that may be evaluated in this article, or claim that may be made by its manufacturer, is not guaranteed or endorsed by the publisher.
